# Antibacterial and antibiofilm potentials of vancomycin-loaded niosomal drug delivery system against methicillin-resistant *Staphylococcus aureus* (MRSA) infections

**DOI:** 10.1186/s12896-024-00874-1

**Published:** 2024-07-08

**Authors:** Jaber Hemmati, Mohsen Chiani, Babak Asghari, Ghodratollah Roshanaei, Sara Soleimani Asl, Morvarid Shafiei, Mohammad Reza Arabestani

**Affiliations:** 1 Department of Microbiology, School of Medicine, Hamadan University of Medical Sciences, Hamadan, Iran; 2 Department of Bacteriology, Pasteur Institute of Iran, Tehran, Iran; 3 Department of NanoBiotechnology, Pasteur Institute of Iran, Tehran, Iran; 4 Department of Biostatistics, School of Public Health, Hamadan University of Medical Sciences, Hamadan, Iran; 5 Department of Anatomy, School of Medicine, Hamadan University of Medical Sciences, Hamadan, Iran; 6 Infectious Disease Research Center, Hamadan University of Medical Sciences, Hamadan, Iran

**Keywords:** Niosome, Biofilm, Methicillin-resistant *S. aureus*, Vancomycin, Drug delivery system

## Abstract

The threat of methicillin-resistant *Staphylococcus aureus* (MRSA) is increasing worldwide, making it significantly necessary to discover a novel way of dealing with related infections. The quick spread of MRSA isolates among infected individuals has heightened public health concerns and significantly limited treatment options. Vancomycin (VAN) can be applied to treat severe MRSA infections, and the indiscriminate administration of this antimicrobial agent has caused several concerns in medical settings. Owing to several advantageous characteristics, a niosomal drug delivery system may increase the potential of loaded antimicrobial agents. This work aims to examine the antibacterial and anti-biofilm properties of VAN-niosome against MRSA clinical isolates with emphasis on cytotoxicity and stability studies. Furthermore, we aim to suggest an effective approach against MRSA infections by investigating the inhibitory effect of formulated niosome on the expression of the biofilm-associated gene (*icaR*). The thin-film hydration approach was used to prepare the niosome (Tween 60, Span 60, and cholesterol), and field emission scanning electron microscopy (FE-SEM), an in vitro drug release, dynamic light scattering (DLS), and entrapment efficiency (EE%) were used to investigate the physicochemical properties. The physical stability of VAN-niosome, including hydrodynamic size, polydispersity index (PDI), and EE%, was analyzed for a 30-day storage time at 4 °C and 25 °C. In addition, the human foreskin fibroblast (HFF) cell line was used to evaluate the cytotoxic effect of synthesized niosome. Moreover, minimum inhibitory and bactericidal concentrations (MICs/MBCs) were applied to assess the antibacterial properties of niosomal VAN formulation. Also, the antibiofilm potential of VAN-niosome was investigated by microtiter plate (MTP) and real-time PCR methods. The FE-SEM result revealed that synthesized VAN-niosome had a spherical morphology. The hydrodynamic size and PDI of VAN-niosome reported by the DLS method were 201.2 nm and 0.301, respectively. Also, the surface zeta charge of the prepared niosome was − 35.4 mV, and the EE% ranged between 58.9 and 62.5%. Moreover, in vitro release study revealed a sustained-release profile for synthesized niosomal formulation. Our study showed that VAN-niosome had acceptable stability during a 30-day storage time. Additionally, the VAN-niosome had stronger antibacterial and anti-biofilm properties against MRSA clinical isolates compared with free VAN. In conclusion, the result of our study demonstrated that niosomal VAN could be promising as a successful drug delivery system due to sustained drug release, negligible toxicity, and high encapsulation capacity. Also, the antibacterial and anti-biofilm studies showed the high capacity of VAN-niosome against MRSA clinical isolates. Furthermore, the results of real-time PCR exhibited that VAN-niosome could be proposed as a powerful strategy against MRSA biofilm via down-regulation of *icaR* gene expression.

## Introduction

*Staphylococcus aureus* is a pathogen that poses a substantial risk to human life and presents significant medical issues for the healthcare system [1–3]. In hospitalized patients, this pathogen can cause a wide range of illnesses with significant mortality rates, such as necrotizing pneumonia and the infection of the respiratory tract, prosthetic joint, and surgical site [4, 5]. The fast spread of multi-drug resistant (MDR) *S. aureus* isolates among infected individuals in recent years has raised serious concerns about public health and enormously complicated treatment options against this organism [6, 7]. The high ability of *S. aureus* to form a biofilm can account for its easy attachment to various biotic and abiotic surfaces in the hospital settings. Moreover, biofilm formation could delay wound healing, causing chronic infections, where 43–88% of *S. aureus* strains are from diabetic and bedsore ulcers [8]. Indeed, biofilm decreases the effective dosage of antimicrobial agents by inhibiting their diffusion into bacterial space and shielding organisms against unfavorable conditions, including excessive antimicrobial agents and host immunity responses [9, 10]. Conversely, biofilm may offer the best conditions for horizontal gene transfer and spreading of drug resistance genes throughout bacterial populations [11].

Methicillin-resistant *S. aureus* (MRSA) strains frequently resist many antibiotics, leading to drastic therapeutic challenges in healthcare settings and communities [12, 13]. These isolates are characterized by the *mecA* gene, which is rapidly transferring among other staphylococcal strains [14]. MRSA isolates are a prominent causative agent in chronic wound infections which have detrimental influences on human quality of life. In this regard, the Centers for Disease Control and Prevention (CDC) reported that, in the United States, the annual (2018) death associated with MRSA was estimated at 20,000, and this mortality rate was greater than other resistant bacterial pathogens [15]. Furthermore, it is proven that MRSA has more ability to form biofilms than methicillin-sensitive *S. aureus* (MSSA), causing MRSA to be known as a significant pathogenic isolate [16]. The MRSA isolates’ ability to build biofilms causes a delay in the re-epithelialization of infected wounds and soft tissues, lengthening the healing period. Moreover, MRSA biofilm has been linked to chronic wounds such as venous ulcers, pressure sores, and diabetic foot ulcers [17].

Vancomycin (VAN), as a glycopeptide antibiotic, inhibits the synthesis of bacterial cell walls, which plays a crucial role in combating Gram-positive bacterial pathogens. VAN targets the bacterial cell wall by attaching itself to the D-Ala-D-Ala terminus of peptidoglycan precursors, which prevents cell wall cross-linking and ultimately resulting in bacterial cell death [18]. In the 1980s, the prophylaxis and treatment of significant infections related to *S. aureus* isolates, particularly MRSA, shifted towards administrating VAN [19]. However, the uncontrolled prescription of glycopeptide antibiotics has resulted in the spread of VAN-resistant *S. aureus* isolates, and an analysis of clinical research published in the literature shows that the susceptibility of MRSA isolates to VAN is rapidly declining globally. This underscores the significant impact of MRSA infections and the urgent need to come up with effective treatment strategies and take prevention measures [20].

Vesicular drug delivery systems were initially introduced by a British scientist, which are composed of concentric bilayer membranes [21, 22]. These systems could be applied to the encapsulation of a wide range of materials and are gaining traction among scientists in biomedical applications [23]. Niosomes are among these vesicular systems that have been presented as powerful drug delivery systems [24]. The bilayer structure of niosomes is composed of amphiphilic molecules, providing several favorable properties. Also, negligible toxicity, biodegradability, non-immunogenicity, bioavailability, structure flexibility, and simple formulation are among the features that improve the pharmaceutical behavior of niosome [25, 26]. However, drug leakage from bilayer membranes is among the main drawbacks of niosomes that could limit their applications in drug delivery [27]. The practical ways to overcome this limitation can include selecting proper non-ionic surfactants, modifying the synthesis methods, and combining the appropriate proportion of ingredients, which considerably impact on niosomal efficiency [28]. Cholesterol is known as a significant membrane additive and could be involved in the niosomal composition, which has a considerable effect on reducing drug leakage from bilayer membrane [29]. In the current research, a nanosized spherical niosome containing VAN at high entrapment capacity was formulated for the gradual drug release from the niosomal system. Furthermore, the physical stability analysis of synthesized nanoparticles could significantly impact the synthesis of the optimized niosome for further biomedical purposes. Several investigations have indicated that niosomal vesicular systems may serve as efficient nano-carriers for medicinal applications, specifically targeting bacterial infections [30]. The antibacterial properties of several niosomal drug delivery systems against different bacterial pathogens, including *Staphylococcus epidermidis*, *Bacillus subtilis*, *Pseudomonas aeruginosa*, *Klebsiella pneumoniae*, *Acinetobacter baumannii*, and *Escherichia coli* were approved [31–33]. Also, the results of some experiments exhibited that niosome nanoparticles, as a powerful drug delivery system, hold promise for solving serious challenges related to *S. auras* [34–36]. However, the present research aims to evaluate the antibacterial activity of VAN-niosome against MRSA clinical strains with emphasis on cytotoxicity study on human foreskin fibroblast (HFF) cell line. Furthermore, the inhibitory and eradication effects of VAN-niosome against MRSA biofilms were assessed. Finally, this study aims to suggest a promising approach against MRSA infections by evaluating the anti-biofilm activities of formulated niosome using real-time PCR.

## Material and method

### Materials

Span 60 (sorbitan monostearate, 98%), cholesterol (≥ 95%), Tween 60 (polyoxyethylene sorbitan monopalmitate, ≥ 98%), chloroform (HPLC grade), methanol (HPLC grade), crystal violet (≥ 90%), antibiotic disks, and all culture media were purchased from Merck, Germany. VAN hydrochloride (≥ 85%), Spectra/ Por^®^ dialysis membrane (MWCO 12–14 KDa), and ultra Amicon tube (cutoff 30 kDa) were also supplied from Sigma-Aldrich, India. The standard MRSA strain, *S. aureus* ATCC 6538, was also provided by the microbiological collection bank of the Pasteur Institute of Iran.

### Bacterial isolation and antibiotic resistance pattern

A total of 36 *S. aureus* isolates were collected from different inpatient wards of the Loghman-e Hakim Hospital, Tehran, Iran, for three months from November 2021 to January 2022. The strains were taken from 75 clinical wound specimens, including diabetic, bedsore, and burn, using sterile cotton swabs. The collected isolates were diagnosed as *S. aureus* by routine microbiological analyses [37, 38]. For screening of MRSA, the antibiotic susceptibility profile for oxacillin (1 µg) and cefoxitin (30 µg) disks was carried out [39]. The polymerase chain reaction (PCR) was also performed for final MRSA confirmation by specific primers for the *mecA* gene [40]. Finally, all MRSA strains were kept in tryptic soy broth (TSB) medium containing 15% glycerol at -20 °C for further analysis.

The disk diffusion method was used to accomplish the antimicrobial susceptibility testing (AST) of *S. aureus* isolates in compliance with the recommended protocols of the Clinical and Laboratory Standards Institute (CLSI) [41]. The following five antimicrobial classes, including aminoglycosides (gentamycin), macrolides (azithromycin, erythromycin), tetracyclines (doxycycline), fluoroquinolones (ciprofloxacin, levofloxacin), and lincosamides (clindamycin) were tested. Moreover, the MDR pattern was identified as the non-susceptibility to at least one agent in ≥ 3 antimicrobial groups [42].

### Screening of biofilm formation

A microtiter plate (MTP) assay was employed to screen the biofilm formation ability of *S. aureus* isolates [43, 44]. In this method, the overnight cultures of tested isolates in a TSB medium containing 1% glucose were adjusted to match a 0.5 McFarland concentration. Afterward, diluted suspensions were dispensed into a 96-well microtiter plate and overnight incubated at 37 °C. After washing the plate, the biofilms were fixed using absolute methanol and were stained using 200 µl of 1.5% w/v crystal violet solution for 15 min. Subsequently, the unbound dye was aspirated, and each well was rinsed in triplicate. After solubilizing the bound stain with 200 µl of 33% v/v acetic acid solution, the optical densities (ODs) of each well were evaluated using a microtiter plate ELISA reader (BioTek, Germany) at 570 nm. Ultimately, the biofilm patterns were divided into four groups using the following formula: OD ≤ ODc (non-adherent), ODc < OD ≤ 2 × ODc (weak biofilm), 2 × ODc < OD ≤ 4 × ODc (moderate biofilm), and 4 × ODc < OD (strong biofilm). Notably, the biofilm pattern of each isolate was screened in triplicate, and *S. aureus* ATCC 6538, as a strong biofilm producer, was applied as positive control.

### Niosomal formulation

In the current study, the thin-film hydration method was applied to formulate VAN-niosome [45]. The mechanism of niosomal drug formation relies on the self-assembly properties of nonionic surfactants and cholesterol in an aqueous environment. In this research, a defined amount of Tween 60, Span 60, and cholesterol (with a molar ratio of 2:2:1) was dissolved in 20 ml 2:1 v/v of chloroform-methanol and magnetically stirred at 150 rpm for 45 min at 25 °C to obtain a uniform solution. After this step, the organic solution was removed under vacuum conditions at 60 °C for 45 min at 120 rpm using a rotary evaporator (WB Eco Laborota 4000 Model, Heidolph Instruments, Germany). The residual solvent was removed by nitrogen gas, and subsequently, the dried lipid was hydrated in 20 ml of PBS (pH ~ 7.4, 100 mM) containing VAN for 45 min. In the next step, the niosomal formulation was sonicated with a probe sonicator (Hielscher UP50H ultrasonic processor, Germany) for 10 min. Finally, the synthesized suspension was visually observed for turbidity and flocculation, and refrigerated for further experimentations. Notably, the blank niosome was formulated using the same protocol without the addition of VAN. Also, the molar ratio of lipid to VAN was adjusted to 20:1 [46].

### Characterization of niosome

#### Morphology, hydrodynamic size, and surface zeta potential

In our study, field emission scanning electron microscopy (FE-SEM) was used to analyze the niosomal morphology [47]. For FE-SEM micrographs, one droplet of prepared niosome (1:100 diluted with deionized water) was mounted on the FE-SEM sample stub and coated with a 200 nm conductive gold layer. The images were analyzed with ImageJ software (bundled with Java version 1.8.0_172) [48]. Additionally, the niosomal physicochemical characteristics, including hydrodynamic size, surface zeta potential, and polydispersity index (PDI), were assessed with the dynamic light scattering (DLS) method using a Zeta-sizer device (Malvern Instrument Ltd. Malvern, UK) at 633 nm. The DLS method was performed in triplicate, and the synthesized niosome was analyzed at the same conditions of pH, concentration, and temperature (7.4, 0.1 mg ml^− 1^, and 25 °C).

#### Entrapment efficiency (EE%)

In the current research, the ultra-centrifugation assay was used to evaluate the EE% of VAN-niosome. Firstly, 2 ml of niosomal formulation was centrifuged at 7,500 g at 25 °C for 25 min in an ultra Amicon tube [49]. Subsequently, the OD of the supernatant was calculated with a UV spectrophotometry (Jasco V-530, Japan) at 281 nm, and the specific amount of free VAN was estimated by a standard curve equation [50]. Finally, the EE% was reported using the following formula: EE% = [(A-B)/A] ×100.

Where A is the specific amount of loaded VAN into niosomal suspension, and B is the specific amount of free VAN in the supernatant.

#### In vitro release

In this study, the dynamic dialysis method was used to determine the release kinetics of the niosomal VAN formulation [51]. Firstly, the un-entrapped VAN was seperated by ultra-centrifugation at 4 °C at 100,000 g for 45 min [52]. Afterward, the dialysis bag containing 1 ml of VAN-niosome was placed in 30 ml of recipient medium (PBS, 5 mM, pH ~ 7.4) and magnetically stirred for 48 h at 37 °C at 150 rpm. Then, 1 ml of recipient media was sampled at intervals of 1, 2, 4, 8, 24, and 48 h, and the specific amount of released drug was assessed using the standard curve equation. Also, an equivalent volume of fresh PBS (5 mM, pH ~ 7.4, 37 °C) was substituted with the aliquoted samples. Notably, to assess the drug release kinetics from a synthesized niosomal system, the data of release analysis were examined through mathematical methods according to the kinetic models’ equations such as Weibull and Hyperbolic Tangent Function, Korsmeyer–Peppas equation, and zero- to fifth-order polynomials [53].

#### Fourier-Transform Infrared spectroscopy (FT-IR)

Any possible interaction between loaded VAN and niosome was examined using a FT-IR spectroscopy instrument (Spectrum Two, U.S.A.), which was carried out in the 400–4000 °C temperature range.

#### Stability studies

To perform the stability analysis, the hydrodynamic size, PDI, and EE% of the synthesized VAN-niosome were assessed for a 30-day storage time at 4 °C and 25 °C.

#### **Cytotoxicity analysis**

The MTT (dimethylthiazol-2-yl)-2, 5-diphenyl-tetrazolium bromide) method was applied to determine the biocompatibility of VAN-niosome against the HFF cell line taken from the Pasteur Institute of Iran, Tehran, Iran [54]. Initially, the HFF cells were cultured in a 96-well microtiter plate at 1 × 10^4^ cells per well, and the plate was overnight incubated with 5% CO_2_ at 37 °C. Then, the increasing concentrations of samples were added to HFF cells, and after 24 h incubation at 37 °C, each well was filled with 15 µl of 5 mg ml^− 1^ MTT dye. Subsequently, the plate was incubated for 4 h at 37 °C, and 200 µl of dimethyl sulfoxide (DMSO) was added to each well. Finally, the ODs were measured using a microplate ELISA reader at 570 nm (29), and the cytotoxicity of VAN-niosome was calculated as the following equation: Cell viability (%) = (OD sample / OD negative control) × 100.

Notably, all assays were performed in triplicate, and the well-containing HFF cell without adding samples was considered a negative control. Also, the HFF cell treated with DMSO was considered a positive control.

### Antibacterial activity of synthesized niosome

The approved CLSI broth microdilution protocol was used to evaluate the minimum inhibition and bactericidal concentrations (MIC/MBC) of formulated niosomes against MRSA strains [55]. Firstly, Mueller-Hinton broth (MHB) medium containing gradient concentrations of sample was dispensed to a 96-well microtiter plate. In the next step, 0.5 McFarland suspensions of MRSA isolates were inoculated into the wells, and the plate was overnight incubated at 37 °C. The minimum sample concentrations without visible bacterial growth were reported as MICs. The MBC was also considered the well containing the lowest concentration with no-growth (> 99%) on the Mueller-Hinton agar (MHA) medium after overnight incubation at 37 °C. All assays were conducted in triplicate, and microbial strain and uninoculated mediums were positive and negative controls, respectively.

### Anti-biofilm activity of synthesized niosome

#### **Biofilm inhibition and eradication**

To evaluate the biofilm inhibitory effect of niosomal VAN, 200 µl of selected bacterial suspensions (10^6^ CFU/ml) diluted with TSB were inoculated into a 96-well microtiter sterile plate. Then, the serial dilutions of sample were added into each well, and the plate was overnight incubated at 37 °C. After washing the wells, the formed biofilms were fixed with absolute methanol and subsequently stained with crystal violet. In the final step, the ODs were estimated with a microplate ELISA reader at 570 nm [56–58].

The anti-biofilm efficacy of synthesized niosome was also examined using minimal biofilm eradication concentrations (MBEC). Firstly, the MRSA strains were allowed to produce 1- and 3-day-old biofilms. Then, the serial dilutions of sample were added to each well, and the plate was incubated at 37 °C overnight. Subsequently, 10 µl of the well’s content was cultured on an MHA medium. After 48 h incubation, the number of bacterial colonies was enumerated, and the lowest concentration killing all embedded bacteria was determined as MBEC. Notably, *S. aureus* ATCC 6538 and the uninoculated medium were used as positive and negative controls, respectively [59].

#### Biofilm gene expression

The impact of niosomal formulation on biofilm gene expression was examined using a real-time PCR assay. At first, the isolates were treated with a sub-MIC concentration of sample. Next, the total RNAs were extracted with an RNX-Plus kit (SinaColon Co, Iran), and reverse-transcribed to cDNA with random hexamer primers. To determine the expression of biofilm-associated gene (*icaR*), real-time PCR was performed with SYBR green qPCR master mix (SinaColon Co, Iran) as follows: a 10-minute initial denaturation at 95 °C, 40 amplification cycles of denaturation at 95 °C for 30 s, annealing at 60 °C for 90 s, extension at 72 °C for 15 s, and a 5-minute final extension at 72 °C. To normalize the expression level of the biofilm gene, the cycle threshold (CT) values of the *icaR* gene were compared with those of *16 S rRNA*, as a housekeeping gene. In the final step, the relative expression of *icaR* gene was calculated according to the ΔΔCT method [60].

### Statistical analysis

The t-test and Chi-squared were applied to compare the investigated parameters with a significance level of less than 0.05. All graphs were created with GraphPad Prism version 9.0.

## Results

### Bacterial isolation and biofilm formation ability

In current research, a total of 36 *S. aureus* strains consisting of 12 (33%) MRSA and 24 (66%) MSSA were isolated from 75 clinical samples. Also, the AST results showed that among 36 *S. aureus* isolates, 20 (55.5%) and 16 (44.4%) strains were MDR and non-MDR, respectively. Furthermore, the MTP method demonstrated that *S. aureus* strains had a high ability to biofilm formation and 32 of 36 (88.8%) isolates formed a biofilm. Also, among 32 biofilm-forming isolates, 15 isolates (46.8%), 8 isolates (25%), and 9 isolates (28.1%) had strong, moderate, and weak biofilm profiles, respectively. Also, 100% and 70.8% of MRSA and MSSA strains were biofilm formers, respectively. Table 1 presents the drug resistance profile and biofilm pattern of 36 *S. aureus* strains in this study.

**Table 1:** Antibiotic resistance and biofilm formation patterns of *S. Aureus* isolates

Strain NO.	Methicillin resistant profile	Biofilm mod	MDR/non-MDR
1	MRSA	Strong	MDR
2	MSSA	Strong	MDR
3	MSSA	Moderate	MDR
4	MSSA	Strong	MDR
5	MRSA	Weak	MDR
6	MSSA	Moderate	non-MDR
7	MRSA	Strong	MDR
8	MRSA	Weak	non-MDR
9	MSSA	-	non-MDR
10	MRSA	Moderate	MDR
11	MSSA	Strong	non-MDR
12	MSSA	-	non-MDR
13	MRSA	Weak	MDR
14	MSSA	-	non-MDR
15	MSSA	Strong	MDR
16	MSSA	-	non-MDR
17	MRSA	Moderate	MDR
18	MSSA	Moderate	non-MDR
19	MRSA	Strong	MDR
20	MSSA	Strong	non-MDR
21	MSSA	Strong	MDR
22	MSSA	-	non-MDR
23	MSSA	Weak	non-MDR
24	MRSA	Moderate	MDR
25	MSSA	Strong	non-MDR
26	MRSA	Strong	MDR
27	MSSA	Weak	non-MDR
28	MSSA	Weak	MDR
29	MSSA	Strong	MDR
30	MSSA	Weak	MDR
31	MRSA	Strong	MDR
32	MSSA	Weak	non-MDR
33	MSSA	-	non-MDR
34	MRSA	Strong	MDR
35	MSSA	Moderate	MDR
36	MSSA	-	non-MDR

MRSA: Methicillin-resistant *S. aureus*, MSSA: Methicillin-sensitive *S. aureus*, MDR: Multi-drug resistant

### Physicochemical characterization of VAN-niosome

#### Morphology, hydrodynamic size, PDI, and surface zeta potential

**Fig. 1:**
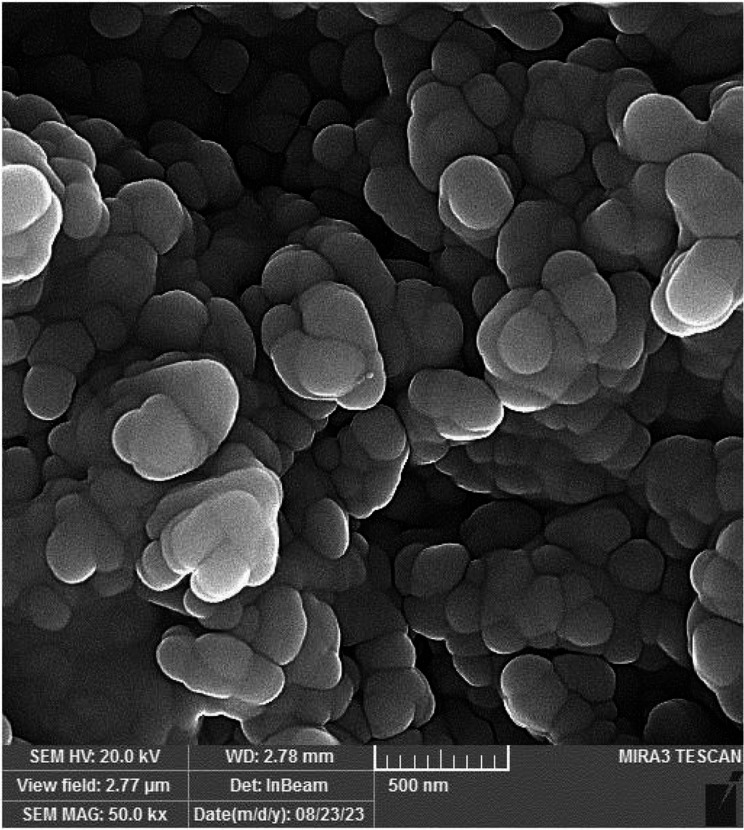
Spherical morphology of VAN-niosome based on the field emission scanning electron microscopy (FE-SEM)

**Fig. 2:**
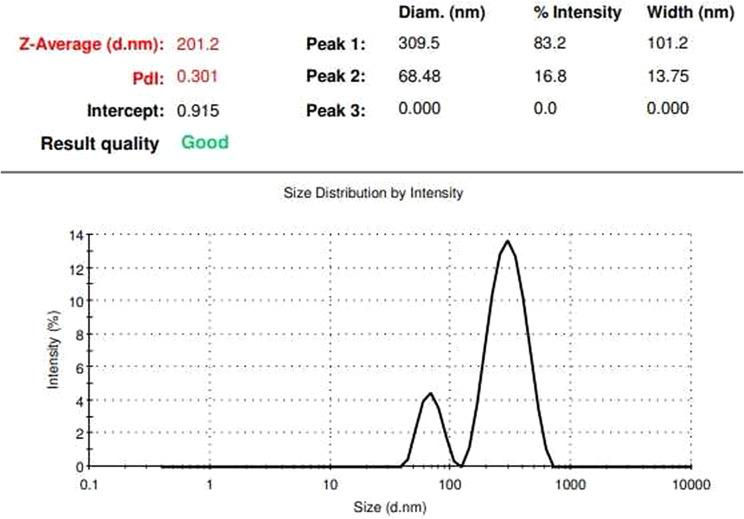
Size distribution curve of VAN-niosomes obtained by the Stokes-Einstein equation [61]

Based on the images taken by FE-SEM, the VAN-niosome had a spherical morphology (Fig. 1). The hydrodynamic size of the VAN-niosome reported by the Zeta-sizer was 201.2 nm. Furthermore, the PDI value was evaluated to be 0.301, representing the acceptable homogenous dispersion for synthesized nanoparticles (Fig. 2). Additionally, the surface zeta charge of the VAN-niosome reported by the DLS method was − 35.4 mV (Table 2).

**Table 2:** Surface zeta potential of VAN-niosomes obtained by the Smoluchowski equation [62]

Result		Mean (mV)	Area (%)	Width (mV)
Zeta Potential (mV): -35.4	Peak 1:	0.00	0.00	0.00
Zeta Deviation (mV): 0.00	Peak 2:	0.00	0.00	0.00
Conductivity (mS/cm): 6.73	Peak 3:	0.00	0.00	0.00
Result quality Good				

#### Niosomal entrapment efficiency (EE%)

In our study, EE% of the niosomal suspension ranged from 58.9 to 62.5%, representing the high potential of niosomes in drug encapsulation. It is proven that incorporation into niosomal formulation could enhance the pharmaceutical activities of loaded drugs, displaying the prominent ability of niosomes to be applied in clinical applications [63].

#### In vitro release profile

**Fig. 3:**
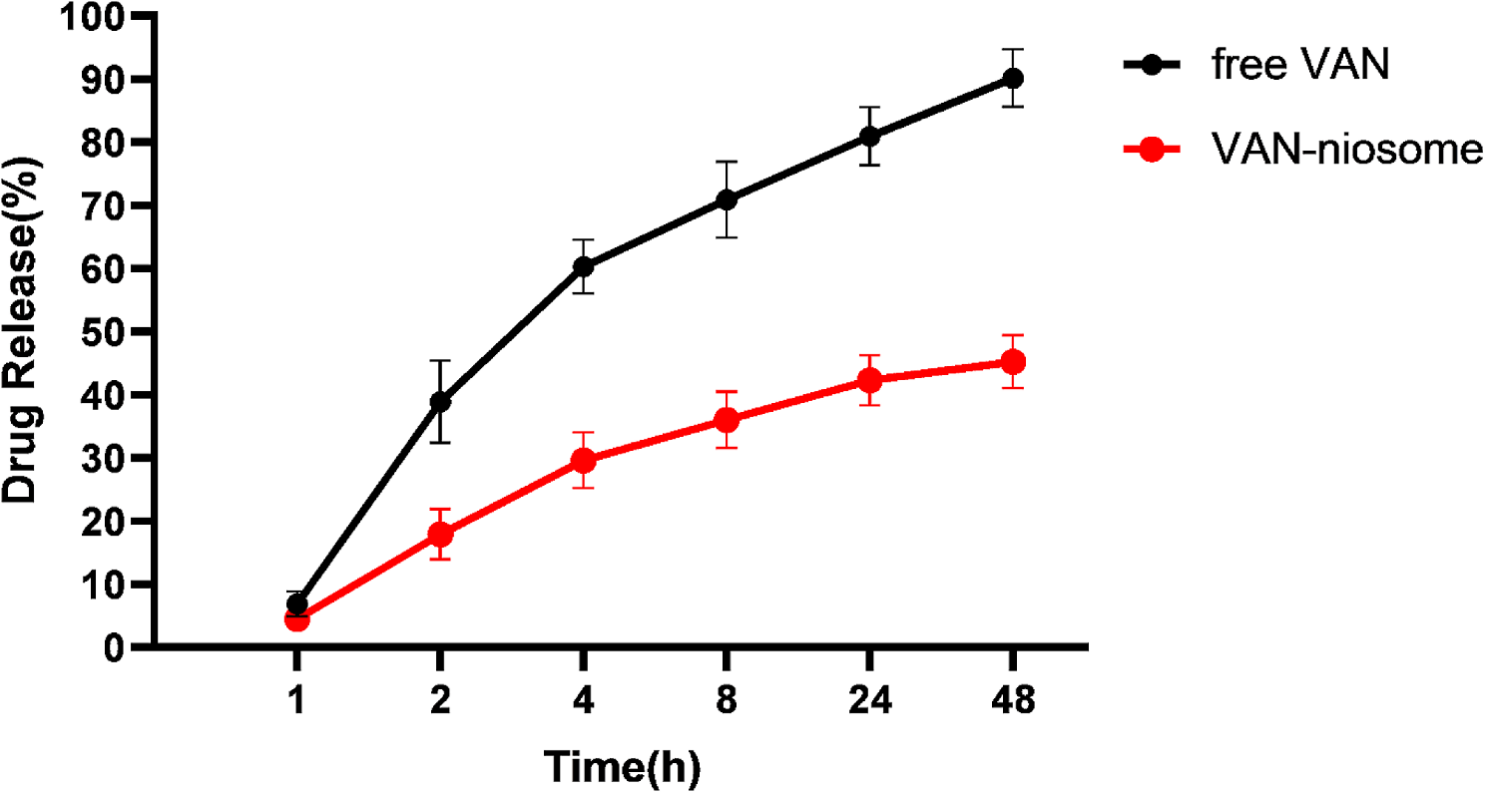
Comparison of in vitro release profiles of free VAN and niosomal VAN at 37 °C

According to statistic evidence, first-order kinetic was the most suitable model for describing the drug release for both free and niosomal formulations. As shown in Fig. 3, approximately 60% of VAN was released from the free formulation in the first 4 h. Whereas, only 30% of incorporated VAN was released from the niosomal suspension during this time. Moreover, within 48 h, around 90% and 48% of drug was released from free and niosomal formulations, respectively. The in vitro drug release study proves that niosomes can be an efficient drug delivery system through preventing burst drug release.

#### FTIR

**Fig. 4:**
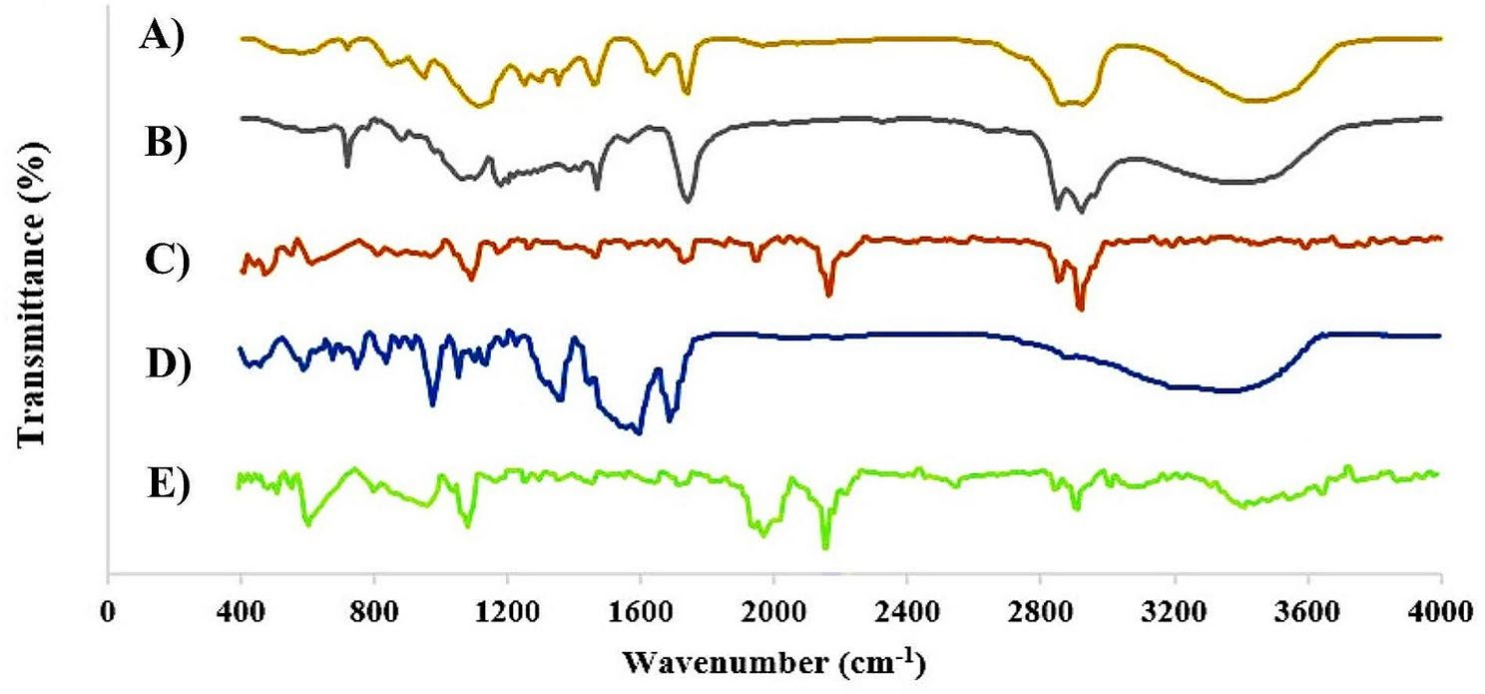
Fourier Transform Infrared (FTIR) spectra of (**A**) Tween 60, (**B**) Span 60, (**C**) VAN, (**D**) niosome, and (**E**) VAN-niosome

FTIR spectrum (Fig. 4) of Span 60, Tween 60, VAN, niosome, and VAN-niosome revealed that the band around 3440 cm^− 1^ is related to O–H stretching of phenolic elements. The band 2933 cm^− 1^ is related to C–H stretching of methylene group in aliphatic hydrocarbons and esters, the band around 2300 is assigned C-O and C = C functional groups, the peak around 1700 cm^− 1^ is related to C = O stretching of fatty acid, esters, ketones, and aldehyde, 1500 cm^− 1^ is assigned to C = C stretching of aromatic compounds, 1462 cm^− 1^ is related to C–O–H in-plane bending of fatty acids and others, 1269 cm^− 1^ is related to C–O stretching of fatty acid and ester, and 1044 cm^− 1^ is assigned to C–O stretching of alcohols [60, 64–66]. The presence of these peaks confirmed that VAN is successfully incorporated in the prepared noisome and was covered by used fatty acid in niosomal composition with functional groups, including ketone, aldehyde, carboxylic acid, and others. Also, the presence of these functional groups presents the stability of the synthesized nanostructure [67].

#### **Physical stability study**

**Fig. 5:**
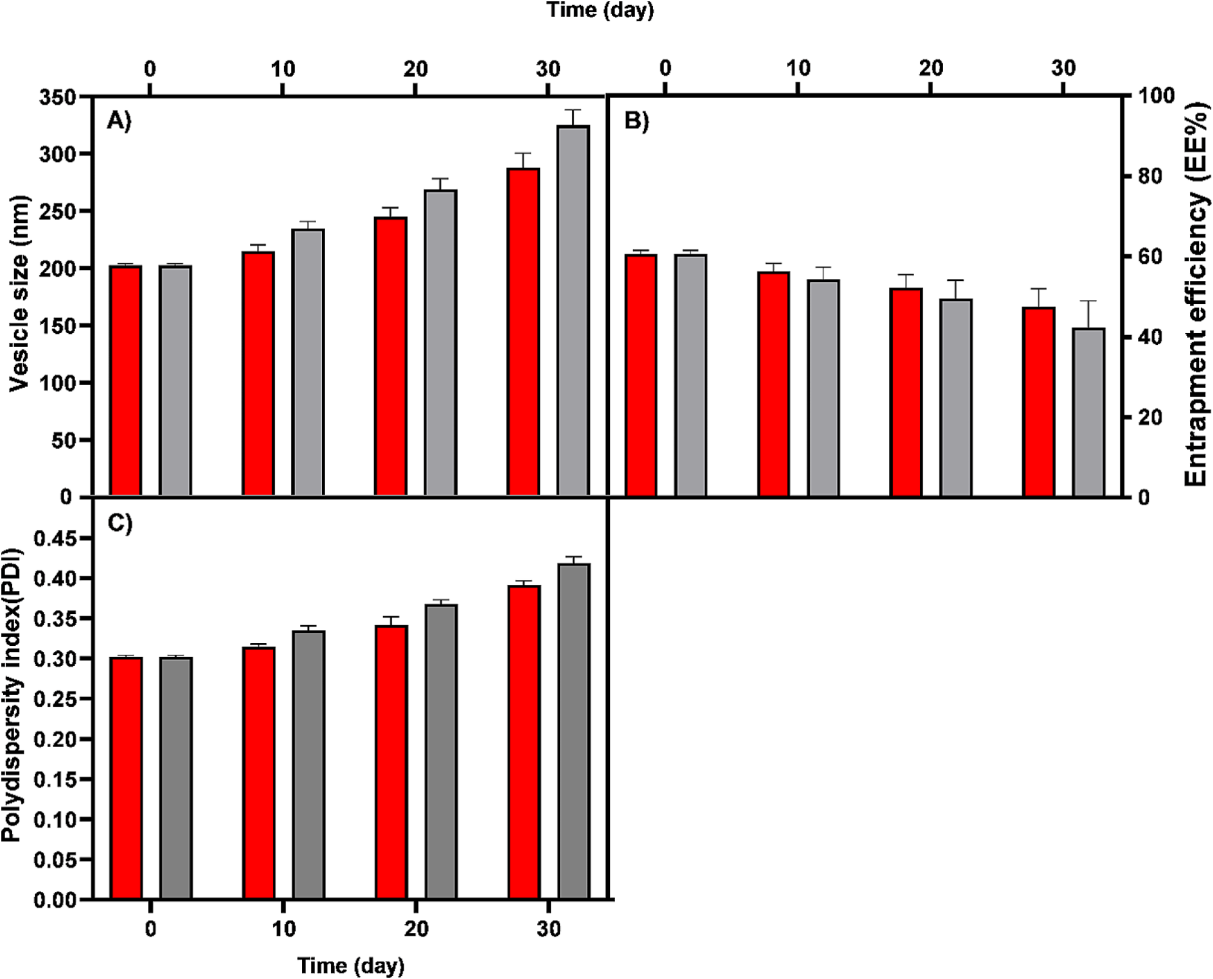
Stability of VAN-niosome stored during a 30-day storage time at 4 °C (Red) and 25 °C (Gray). (**A**) hydrodynamic size, (**B**) entrapment efficiency (EE%), (**C**) polydispersity index (PDI)

In the current research, the stability of the formulated VAN-niosome at 4 °C and 25 °C was analyzed, and its physicochemical properties including hydrodynamic size, PDI, and EE%, were evaluated during a 30-day storage time. As can be seen in Fig. 5, the niosomal VAN formulation kept at 4 °C had less change in hydrodynamic size, PDI, and EE% than the formulation kept at 25 °C, and had more physical stability under these conditions.

#### **Cytotoxicity analysis**

**Fig. 6:**
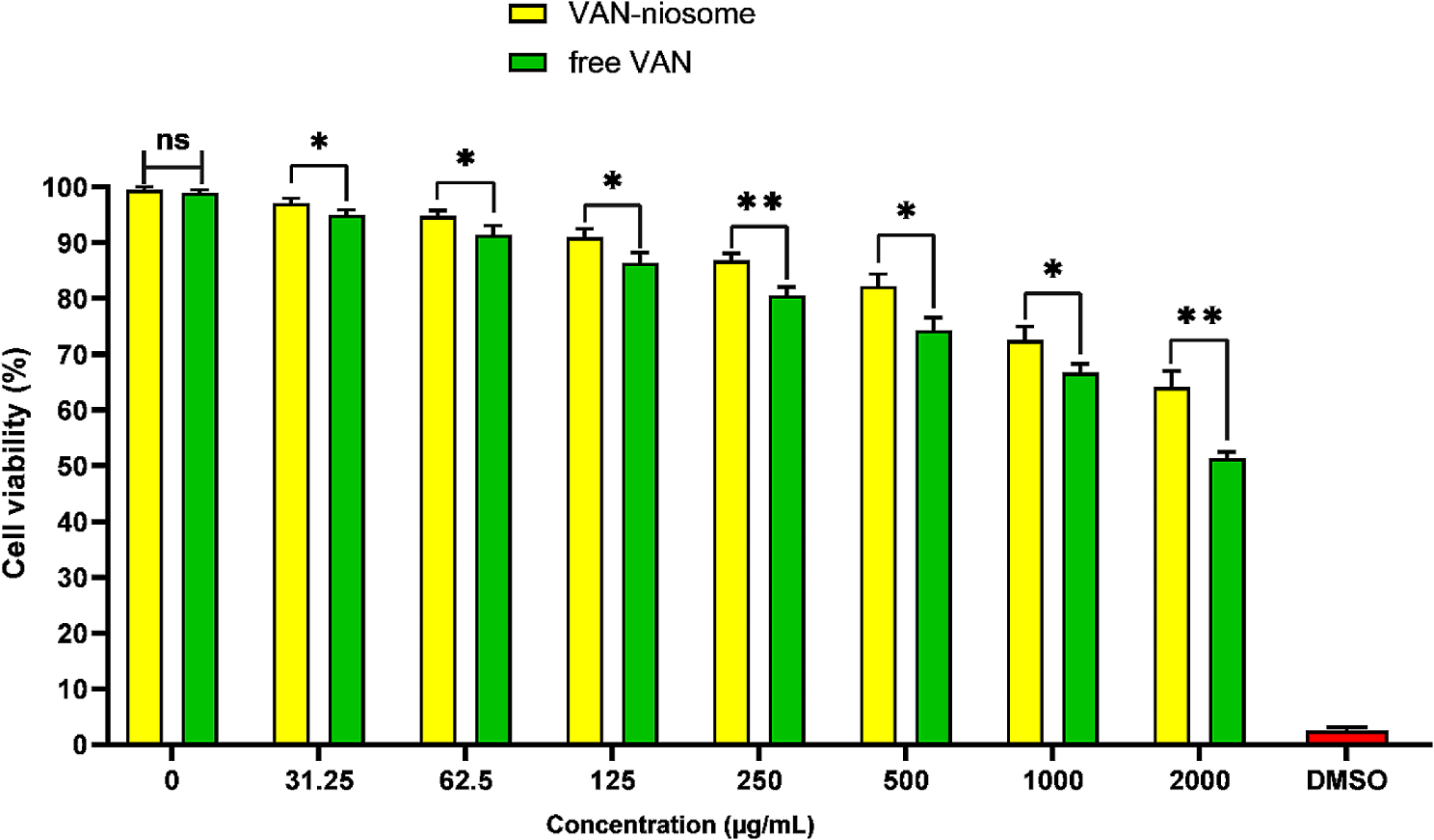
Cell viability of VAN-niosome and free VAN on HFF cell line (mean ± SD, *n* = 3, ns: not significant, *: *p* < 0.05, **: *p* < 0.01)

The cell viability of VAN-niosome and free VAN in different concentrations was evaluated on HFF cell line (Fig. 6). The MTT assay revealed that the cell viability induced by niosomal formulation was significantly higher than that of free VAN in all investigated concentrations. In addition, the cytotoxicity of bare niosomes was investigated, and any significant toxicity was exhibited on HFF cells.

### Antibacterial and antibiofilm analysis

#### MICs and MBCs

**Fig. 7:**
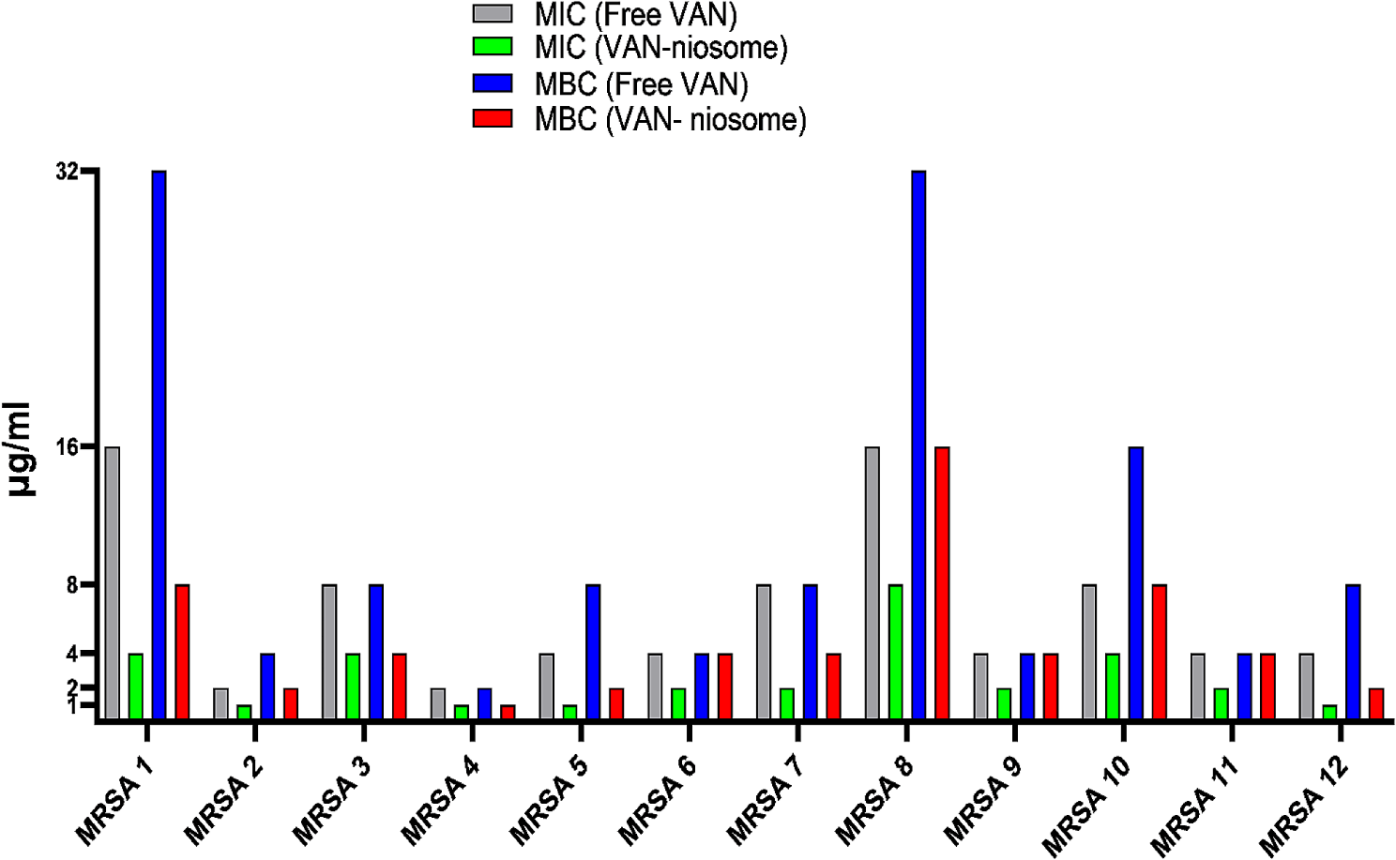
Comparison of antibacterial effect of VAN-niosome with free VAN against MRSA strains

The MICs and MBCs of niosomal VAN formulation against 12 MRSA clinical isolates were analyzed and compared to that of the free VAN (Fig. 7). Our results exhibited that niosoml formulation reduced the MICs of all MRSA isolates by 2-4-fold in comparison to non-niosomal formulation. Also, the VAN-niosome had high bactericidal efficacy, which decreased the MBCs by 2-4-fold against 9 of 12 MRSA isolates. Moreover, the antibacterial ability of the blank niosome was investigated, and no antibacterial activity was found against all MRSA isolates.

#### Biofilm inhibition

**Fig. 8:**
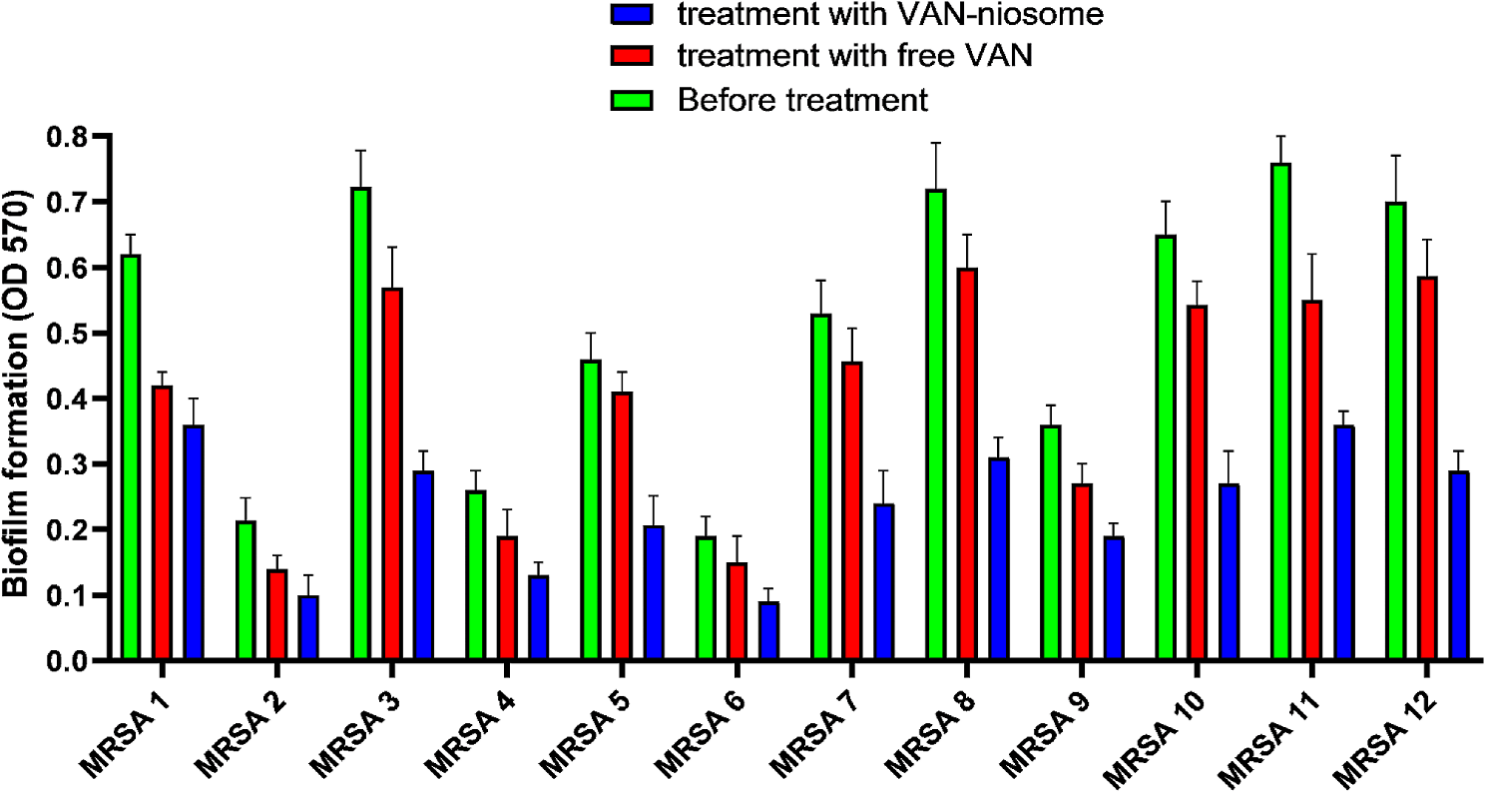
Comparison of anti-biofilm ability of niosomal VAN with free VAN against MRSA strains

In our research, the inhibitory activity of VAN-niosome against MRSA biofilms was assessed and compared with free VAN. The MTP assay revealed that treatment of MRSA isolates with niosomal VAN formulation caused more inhibition in biofilm formation against all MRSA isolates in comparison to the free VAN (Fig. 8). Notably, the anti-biofilm ability of bare niosomes was analyzed, which had any anti-biofilm effect against all tested isolates.

#### **Biofilm eradication**

The anti-biofilm ability of VAN-niosome against MRSA isolates was examined by the MBEC method and compared with free VAN (Table 3). According to our results, the VAN-niosome decreased the 1-day-MBECs against all tested isolates by 2–8-fold in comparison to free VAN. Additionally, the MBEC method exhibited that VAN-niosome eradicated 3-day-old MRSA biofilms at lower concentrations in comparison to non-niosomal formulation. Notably, the anti-biofilm of bare niosomes was investigated, which failed to eradicate any bacterial biofilms at the same concentrations of VAN-niosomal formulation.

**Table 3:** Comparison of minimum eradication concentrations (MBECs) of VAN-niosome with free VAN against MRSA isolates

Isolates No.	Free VAN		VAN-niosome		
	1-day-biofilm	3-day-biofilm	1-day-biofilm	3-day-biofilm	
MRSA1	1024	> 1024	512	1024	
MRSA2	32	64	16	32	
MRSA3	1024	> 1024	128	512	
MRSA4	128	256	32	64	
MRSA5	512	1024	128	512	
MRSA6	64	128	16	32	
MRSA7	512	1024	128	512	
MRSA8	1024	> 1024	512	> 1024	
MRSA9	256	512	128	1024	
MRSA10	1024	> 1024	256	1024	
MRSA11	1024	> 1024	512	> 1024	
MRSA12	1024	> 1024	512	> 1024	

#### **Biofilm gene expression**

**Fig. 9:**
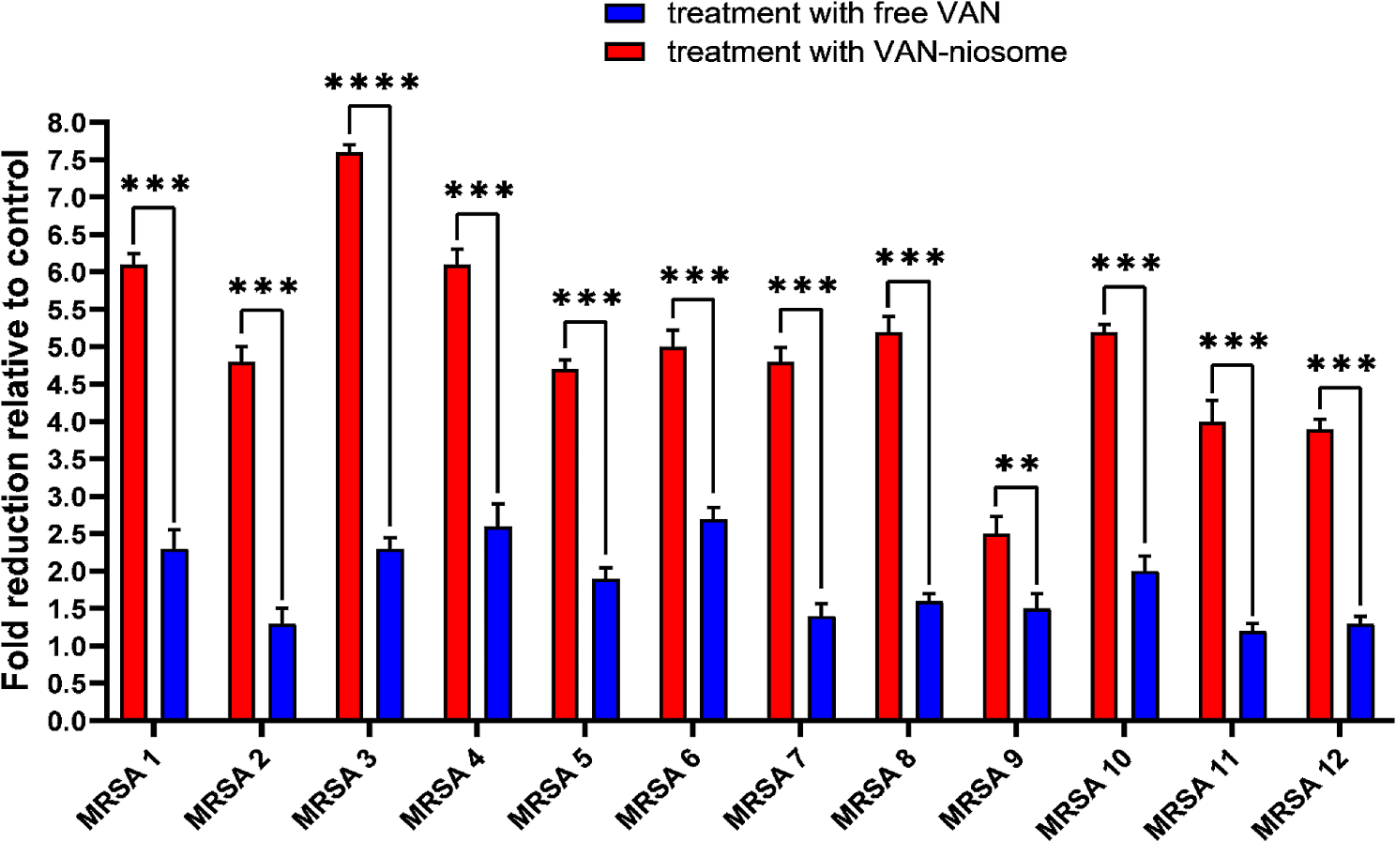
Effect of free VAN and VAN-niosome on biofilm gene expressions against MRSA isolates (mean ± SD, *n* = 3, **: *p* < 0.01, ***: *p* < 0.001)

To further confirmation of the anti-biofilm effectiveness of VAN-niosome, after treating MRSA isolates with sub-MIC concentrations of niosomal VAN and free VAN, the mRNA levels of the biofilm-related gene (*icaR*) were examined using real-time PCR (Fig. 9). Our research revealed that the expression levels of the *icaR* gene in all MRSA isolates were significantly reduced following treatment with niosomal VAN compared with free drug, suggesting the anti-biofilm potential of synthesized formulation against MRSA biofilms.

## Discussion

MRSA is known as one the seriously life-threatening agents with high challenges in hospitalized patients [68, 69]. MRSA has a higher ability to biofilm formation than MSSA isolates, which has a substantial role in emerging drug resistance [16]. In this regard, the present study demonstrated that 100.0% and 70.8% of MRSA and MSSA strains produced biofilm, respectively. The results of our study were in agreement with other studies [60, 70–72], where MRSA strains had a superior tendency to produce biofilms compared to MSSA isolates. Biofilm formation is well-known as a prominent factor in developing MDR strains, posing a significant risk of chronic and recurrent infections [44, 73]. Also, our investigation revealed that all MDR isolates were biofilm formers, indicating a strong association between MDR pattern and biofilm production (P-value < 0.001). Our findings were in agreement with the results of Kwon et al. [71] and Neopane et al. [74], where biofilm production was considerably greater in MDR strains. The explanation for the emergence of MDR pattern in biofilm-forming organisms is attributed to intimate cell-to-cell contact inside the biofilm area which promotes the exchange of plasmids carrying drug-resistance genes [74]. Nevertheless, a different published investigation revealed that the adhesion profile and MDR isolates did not significantly correlate [75]. Also, it is proven that additional factors, including the organisms’ genetic make-up, geographic origin, types of specimens, surface adhesion properties, availability of nutrients [76], efflux pumps [77], and toxin systems [78], contributed to developing drug resistance. Nevertheless, further investigations are needed to clarify the exact correlation between biofilm formation and MDR pattern in a larger numbers of *S. aureus* isolates.

The therapeutic efficacies of a niosomal drug delivery system encapsulating various antibacterial agents rely on designing a formulation with high physical stability [79]. In this study, a stability analysis for a 30-day storage time was performed to investigate the ability of synthesized VAN-niosome to maintain its physicochemical attributes. The physical appearance of prepared niosome was visualized unchanged within 30 days, and neither sedimentation nor flocculation was seen. In addition, the stability analysis revealed that refrigerated niosomes had slower changes in hydrodynamic size, PDI, and EE% in comparison with those kept at room temperature. Moreover, comparing our findings with several studies showed that synthesized niosomes with long-chain non-ionic surfactants (Tween 60 and Span 60) provided greater satisfactory stability than those formulated with Span 40 and Tween 40 [34, 66]. Notably, the findings of in vitro release analysis exhibited that our niosomal formulation had a greater sustained-release pattern compared with other formulations [34, 80]. These superiorities could be discussed that the proper proportion of niosomal ingredients could be a reason for slow-release pattern and better stability of noisome. Additionally, the presence of cholesterol and binary non-ionic surfactants (Tween 60 and Span 60) could reduce the drug leakage from niosomal formulation by increasing bilayer membrane rigidity [81]. Therefore, choosing appropriate surfactants and also luminating the exact amount of niosomal components must be considered as prominent parameters for developing an optimized formulation [82].

Drug resistance in MRSA isolates has been increasing quickly in medical settings in recent years, bringing significant financial challenges to healthcare budgets [83]. Due to some drawbacks, conventional methods have proven difficult to treat MRSA infections effectively [84]; thus, it is critically necessary to devise a novel technique for successfully eliminating the therapeutic obstacles associated with this superbug [27]. Niosome, an influential vesicular drug delivery system, transmits the encapsulated drug into bacterial cells, strengthening the efficacy of anti-MRSA agents [80, 85]. In our study, the anti-MRSA effects of VAN-niosome were investigated, and it was approved that the niosomal delivery system has high potential as an alternative approach against MRSA isolates. Also, the present research showed that encapsulating into niosomal formulation could reduce the MIC and MBC concentrations of free VAN by 2-4-fold (for two comparisons), showing the high ability of niosomal VAN as a powerful antibacterial delivery system. Moreover, in another study by Ghafelehbashi et al. [64] the inhibitory ability of niosomal cephalexin against *S. aureus* strains was found, where the incorporation into niosome reduced the MIC values of free cephalexin by 4-fold. Also, Jastish et al. [80] exhibited the increased anti-*S. aureus* behavior of fluoroquinolones-loaded niosome and the niosomal formulation had a lower MIC concentration (at least 4-fold) in comparison with free drugs. In addition, Heidari et al.‘s study [67] proved that the MIC value of tannic acid-loaded niosome was 2-fold lower than the free drug, indicating niosomal formulation was more potent against *S. aureus* compared with free tannic acid. Additionally, Rezaeiroshan et al. [86] showed the bacteriostatic and bactericidal activities of Trans-ferulic acid-loaded niosome against *S. aureus* isolates, and both MIC and MBC parameters of free drug were decreased by 2-fold via encapsulating into niosome. The results of mentioned researches were in line with our outcomes, proposing that niosomal encapsulation through enhancing the effective dose of encapsulated drugs can be designed for drug delivery against *S. aureus* isolates, including MRSA. The improved antibacterial effect of niosomal encapsulation is supported by this hypothesis that pharmaceutics efficacy of loaded agents would be increased by controlled-release profile from niosomal formulation [80, 87]. In addition, it is hypothesized that niosomes could interact with the peptidoglycan bacterial layer, increasing the permeability of adequate drug concentration into sub-cellular space [88]. Also, the sustained drug release from niosomal vesicles can lead to fewer drug intake intervals, and could be an ideal solution for preventing the resistance mechanism in chronic MRSA infections [89].

As biofilm is recognized as a substantial contributor to the increased pathogenesis of *S. aureus* strains, the early inhibition of bacterial attachment and destruction of formed biofilm on various medical surfaces have become big challenges in healthcare systems [74, 90]. In the present research, the inhibitory activity of VAN-niosome on MRSA biofilms was investigated, and it was demonstrated that entrapment into niosome could significantly decrease the MBEC levels (2-8-fold) of free drug against all MRSA isolates. In agreement with our result, Kashef et al. [91] approved that niosomal ciprofloxacin had a lower MBEC value by 2–4-fold against MRSA isolates in comparison with the free formulation. In another confirmatory study [89], the anti-biofilm ability of niosomal incorporation was confirmed against *S. aureus* strains, where the bacterial attachment to abiotic surface was considerably prevented via covering with encapsulated drug. In addition, the efficacy of cefazolin-loaded niosome on MRSA biofilm eradication was found by Zafari et al. [63], where niosomal formulation had a greater biofilm elimination rate (4-8-fold) than the free drug. Moreover, Shadvar et al. [92] showed the anti-biofilm efficacy of niosomal amoxicillin against MRSA strains, and it was represented that encapsulating into niosomal system greatly reduced (2-4-fold) the density of produced biofilm at same concentration of free amoxicillin. It could be concluded that niosomes inhibit the biofilm formation and efficiently deliver the incorporated contents into the embedded bacteria through facilitating their diffusion into the biofilm structure. Furthermore, durable accessibility of loaded drugs in biofilm matrix can prevent the resistance mechanisms in biofilm-forming bacteria, which could be improved via sustaining drug release pattern of niosomes [29]. The antibiofilm superiority of niosomal formulation is supported by another hypothesis which niosome, as a physical barrier, competes with biofilm-producing bacteria on surface adhesion and significantly contributes to the inhibition of biofilm formation through the reduction of bacterial attachment [93–95]. Furthermore, our investigation demonstrated the down-regulation of biofilm gene (*icaR*) expression relative to the free formulation, validating the biofilm inhibitory effect of VAN-niosome using real-time PCR. This finding was confirmatory with the Mirzaie et al.‘s study [60], where ciprofloxacin-loaded niosome inhibited MRSA biofilm formation, which could be effective on the biofilm genotypic profile through downregulating the expression of the biofilm-associated gene. Furthermore, Heidari et al. [67] showed that niosomal tannic acid significantly reduced the expression of the *S. aureus* biofilm gene when compared to free drug. These findings suggest that niosomes may interact with transcription factors involved in the expression of biofilm-associated genes. Furthermore, the generated reactive oxygen species (ROS) may interact with cellular proteins, including translation and transcription factors, through delivering of the incorporated drugs to the embedded bacteria. Therefore, due to significant potential of MRSA strains in biofilm formation, niosomal drug delivery system could be presented for fighting the therapeutic challenges related to this superbug. However, perspective studies should be designed to find the exact mechanisms of niosomes on anti-biofilm activity of encapsulated contents for further development in medical settings.

## Conclusion

According to this research, niosomal encapsulation improved the pharmaceutical index of free drug, which can be proposed for delivering antimicrobial agents. In addition, incorporation into niosomal system increased the antibacterial effect of free VAN, which could be developed as an effective drug delivery system against MRSA clinical isolates with negligible cytotoxic effect. Also, the results of real-time PCR exhibited that niosomal drug delivery system enhanced the anti-biofilm ability of free VAN against MRSA clinical isolates and could reduce the biofilm-related challenges in health-care systems. However, in vivo studies should be performed to examine the antibacterial and anti-biofilm roles of niosome, which can provide useful insights for the clinical development of this drug delivery system against bacterial infections.

## Data Availability

No datasets were generated or analysed during the current study.
